# The outcomes of home treatment for borderline personality disorder

**DOI:** 10.1192/pb.bp.115.052118

**Published:** 2016-12

**Authors:** Sibel Turhan, Mark Taylor

**Affiliations:** 1 NHS Lothian, Edinburgh, UK

## Abstract

**Aims and method** There is currently no trial or other scientific evidence informing the efficacy of any crisis intervention for people with borderline personality disorder (BPD). We aimed to assess the patterns of service use by patients with BPD taken on for crisis resolution and home treatment between 2010 and 2013. Patients with a diagnosis of BPD were identified and demographic and clinical data were collected.

**Results** All patients were female, and a high proportion had recurrent presentations to crisis and home treatment services in Edinburgh. Many appeared to benefit from intensive home treatment, as measured by the Clinical Global Impression scale. A small number of patients (*n* = 5) were responsible for more than half of all referrals. Polypharmacy, or regular use of multiple medications, was common, with 62% of all patients receiving three or more regular medications.

**Clinical implications** Crisis and home treatment services can be beneficial to most people with BPD in crisis. The high rate of polypharmacy seen in this study is of concern.

Borderline personality disorder (BPD) is a complex and severe mental disorder that manifests as a pervasive pattern of instability in interpersonal relationships and self-image, mood disturbance, impulsive behaviours and repeated self-injury, and dissociation or quasi-psychotic experiences.^1,2^ It is also associated with substantial impairment of social, psychological and occupational functioning and quality of life.^2^ People with BPD are particularly at risk of suicide, with completed suicide occurring in 8–10% of individuals with this disorder, a rate that is approximately 50 times higher than the general population.^1^

BPD is the most common personality disorder seen in clinical settings. It is present in 10% of out-patient mental health clinics, 15–20% of psychiatric in-patients, and 30–60% of clinical populations with a personality disorder. It occurs in an estimated 2% of the general population and has an estimated gender ratio of more than 3:1 for women/men.^1^

The extent of the emotional and behavioural problems experienced by people with BPD varies considerably. Some people with BPD are able to sustain some relationships and occupational activities. Others, with more severe BPD, experience very high levels of emotional distress. They have repeated crises, which can involve self-harm and impulsive aggression, and can have high rates of comorbidity, including addictions. Despite this, people with BPD can be difficult to engage in treatment and frequently present to health services in crisis.^2^ Because of the nature and potential consequences of these crises, the identification and utilisation of effective crisis management interventions with this population is of considerable importance.^2^

The Department of Health in England and Wales has recommended crisis resolution and home treatment (CRHT) in its best practice and policy implementation guides since 2002,^3^ and in 2007 described CRHT as a key step in implementing the National Service Framework, partly to ensure in-patient care was used only where necessary.^3^ National Health Service (NHS) services in Scotland were not constrained by the National Service Framework and did not incorporate functionalised teams such as assertive outreach, early intervention and CRHT teams until 2008.

In this observational study, we evaluated the current patterns of service use in patients with BPD taken on by Edinburgh Intensive Home Treatment Team (IHTT), which is a CRHT that facilitates early discharge from hospital as well as providing intensive home-based care. It was established in 2008 and has been linked to a reduction in psychiatric admissions and positive feedback.^2–4^

## Method

Data were collected during a retrospective examination of medical records of all patients who had primary ICD-10 diagnosis of BPD (code F60.3) taken on by Edinburgh Intensive Home Treatment Team (IHTT) between 2010 and 2013 (4 years). Using unique patient identifiers, each included e-case record was reviewed using *a priori* criteria. IHTT records the severity of the presenting mental disorder using the Clinical Global Impression Scale (CGI)^2^ at admission and clinicians note the improvement or lack thereof in the presenting condition via the CGI-I score at the point of discharge from IHTT. Prospective approval for this service evaluation project was obtained from the local clinical governance department.

The CGI has two components: the CGI-Severity (CGI-S), which rates illness severity, and CGI-Improvement (CGI-I), which rates change from the initiation of treatment (baseline). CGI-S is rated on the following 7-point scale: 1 normal/not at all ill, 2 borderline mentally ill, 3 mildly ill, 4 moderately ill, 5 markedly ill, 6 severely ill, 7 among the most extremely ill patients. CGI-I is similarly rated on a 7-point scale: ‘compared with the patient's condition at admission, this patient's condition is' 1 very much improved, 2 much improved, 3 minimally improved, 4 no change, 5 minimally worse, 6 much worse, 7 very much worse.^3^

Data collected included:

- patients' age and gender
- any medical comorbidities
- currently prescribed medications
- source and reason for referral to IHTT
- the nature of the crisis intervention by IHTT
- duration of treatment by IHTT
- CGI scores on entrance to and at exit from IHTT
- subsequent service provider at discharge from IHTT
- number of repeat referrals/contact with the same patient (i.e. frequency of any IHTT referral).

Each patient's regular medications were reviewed and classified into six groups: antidepressant drugs (selective serotonin reuptake inhibitors (SSRIs), serotonin and noradrenalin re-uptake inhibitors (SNRIs) and tricyclics (TCAs)), antiepileptic drugs used as mood stabilisers, antipsychotic drugs (first- and second-generation antipsychotics (FGAs and SGAs)), anxiolytics (benzodiazepines) and substitute treatment for opioid dependency (methadone).

### Statistical analysis

Pearson χ^2^ with Yates correction was used to test the association between CGI score and hospital admission. An unpaired *t*-test of the means of two groups was also used (with the assumption of normality in CGI distribution being satisfied) to test whether there was a statistically significant reduction in CGI for those who were successfully discharged from IHTT compared with those who needed hospital admission.

## Results

The total number of referrals for the 4-year period was 64, and 100% were female. The median age of patients was 39 years (Table 1).

**Table 1:** Demographic data by year

	2010	2011	2012	2013
Number of BPD patients	20	21	8	15

Median age, years	40.5	39	38.5	45

Total number of IHTT patients	1374	1318	1344	1284

BPD, borderline personality disorder; IHTT, intensive home treatment team.

Of the 64 referrals, the number of individual patients was only 27, as some patients required multiple contacts with the IHTT. Thirteen patients were responsible for 50 referrals (78% of all referrals). A small number of patients (*n* = 5) presented to emergency services frequently owing to repeated crises and suicidal thoughts – they were responsible for 53% (*n* = 34) of all referrals. These five patients were all women who had a traumatic background and multiple prior hospital admissions.

The number of patients who had only one-off contact with IHTT fluctuated from 10% of all referrals in 2011 to 50% of all referrals in 2012, as depicted in Table 2.

**Table 2:** Frequency of contact by year

	2010	2011	2012	2013
Referrals, *n* (patients)	20 (12)	21 (9)	8 (6)	15 (11)

Patients with one-off contact, *n*	7	2	4	8

Patients with ⩽3 contacts in the same year, *n*	2	3	–	1

Patients with previous contacts with IHTT since 2010, *n*	–	5	4	4

IHTT, intensive home treatment team.

There were two completed suicides from the cohort of 27 patients during this period. Both patients were previously known to IHTT but at the time of the suicide were not under the team's care. The first suicide took place in 2013 when the patient was being seen by the local community mental health team, while the second suicide took place in hospital following a lengthy admission.

The majority of referrals (71%) were from the community owing to deterioration in mental health and increased suicidal behaviour; 66% (community referrals and ward referrals for early discharge) were discharged back into community following IHTT interventions. However, for the remaining 34% of referrals IHTT intervention was not enough to manage suicide risk and hospital admission was required (Table 3), whereas 41% of admissions (9 patients) were re-admitted owing to failed early discharge attempt despite IHTT support.

**Table 3:** Referrals and outcome

Year	2010	2011	2012	2013
Reason for referral, *n*				
Deterioration in mental health	11	14	8	10
Facilitating early discharge	8	6	–	5

Source of referral: community, *n*	12	15	8	11

Early discharge	8	6	–	4

Outcome of patients from IHTT				
Discharge home	13	13	7	9
Hospital admission	7	8	1	6

IHTT, intensive home treatment team.

The mean value of CGI of all patients at admission to IHTT was 3.3 (and similarly the mean CGI of the five patients who needed frequent IHTT intervention was 3.2). Using the CGI, an improvement in mental state and behaviour was documented in the majority of patients. The mean CGI improved from a baseline of 3.3 to 2.4 at the time of IHTT discharge in 39 patients. Twenty-two patients were admitted to hospital despite IHTT, with a mean baseline CGI of 4.2. There are significant associations between a lack of improvement as measured by CGI, and hospital admissions (*P*<0.0001; χ = 16, d.f. = 1 with Yates correction), as illustrated in Table 4.

**Table 4:** Clinical Global Impression (CGI) scale improvement in patients with and without hospital admission and IHTT involvement

CGI score	Admitted	Not admitted	Total
1 or 2: improved, *n*	1	25	26

3 or more: not improved, *n*	21	17	38

	22	42	64

IHTT, intensive home treatment team.

Further analysis comparing those who needed hospital admission (mean CGI pre- and post-IHTT involvement: 3.6 and 4.2 respectively) with those who were successfully discharged from IHTT (mean CGI value pre- and post-IHTT: 3.2 and 2.4, unpaired *t*-test) showed a significant difference in CGI between those admitted to hospital and those who were not admitted (two-tailed *P*<0.0001 and 95% CI 1.4 to 2.2).

Polypharmacy is common among this group of patients: 44% of patients who were referred to IHTT were prescribed four or more associated regular medications and 68% were prescribed three or more regular medications (Table 5).

**Table 5:** Classes of medications

	*n* = 20	*n* = 21	*n* = 8	*n* = 15
Year	2010	2011	2012	2013

SSRIs/SNRI, *n*	16	11	4	10

Mood stabiliser, *n*	5	4	4	3

SGA, *n*	11	13	5	9

FGA, *n*	4	7	1	6

Benzodiazepines, *n*	12	11	5	8

Methadone, *n*	–	–	1	2

Regular medications, mean	3.2	3.3	3	3.3

FGA, first-generation antipsychotic; SGA, second-generation antipsychotic; SNRI, serotonin and noradrenalin re-uptake inhibitors; SSRIs, selective serotonin reuptake inhibitors.

## Discussion

A systemic review by Borschmann *et al*^5^ found no randomised controlled trials comparing crisis intervention for people with BPD with usual care, no intervention or waiting list controls. There is currently no adequate evidence base to support any specific crisis intervention for people with BPD.

In this observational study, IHTT's interventions were recorded as ‘increased support’, referring to a combination of brief psychosocial interventions, behavioural activation, judicious use of medication, and/or facilitating immediate access to appropriate services (such as health, housing or legal advice) to alleviate distress. There is no identified specific crisis intervention for this population. In the majority of cases IHTT interventions were successful as the mean CGI improved from baseline 3.3 to 2.4 in those patients who were discharged back into community. The remaining 34% of referrals required hospital admission despite IHTT interventions, with almost 41% needing re-admission owing to failed early discharge despite IHTT intervention.

Relatively few patients (18%, *n* = 5) were responsible for the majority of referrals to IHTT (51%), and were all women in their mid-40s (apart from one) who were regularly prescribed at least four different psychotropic medications. They all had a traumatic background and multiple prior hospital admissions. Interpersonal difficulties (mostly among family members) were usually noted as the main precipitating factor for crisis and increased suicidal thoughts. This small group of individuals were responsible for 50% of all the BPD hospital admissions during this period, illustrating the challenges of working with this population, and the disproportionate demand on the NHS. IHTT interventions and even hospital admissions were not always effective as patients were frequently referred back to IHTT, or indeed later re-admitted to hospital. The reasons for this are unclear, but may include only partial adherence to or rejection of therapeutic interventions.

There has been much debate on the effectiveness of pharmacotherapy in treating different facets of BPD.^6–9^ Several guidelines recommend the use of medications for the treatment of core symptoms of BPD, despite concerns raised regarding the strength of evidence for these recommendations.^4^ The American Psychiatric Association (APA) guidelines, and Abraham & Calabrese, recommend the use of SSRIs as first-choice treatment for affective dysregulation and impulsive-behavioural symptoms, and low-dose antipsychotics for cognitive-perceptual symptoms in BPD.^4–6^ However, Leib *et al*^8^ argue that the previous guidelines were based on research published only up to 1998 and recommend using anticonvulsants for affective dysregulation symptoms and impulsive-behavioural symptoms, and antipsychotics for cognitive-perceptual symptoms.^7^ Nevertheless, despite considering similar evidence to Leib *et al*, the National Institute for Health and Care Excellence (NICE) guidelines draw different conclusions and make clear recommendations against the use of any regular pharmacotherapy in this group:

‘Drug treatment should not be used specifically for borderline personality disorder or for the individual symptoms or behaviour associated with the disorder (for example, repeated self-harm, marked emotional instability, risk-taking behaviour and transient psychotic symptoms). Antipsychotic drugs should not be used for the medium- and long-term treatment of borderline personality disorder. (…) Short-term use of sedative medication may be considered cautiously as part of the overall treatment plan for people with borderline personality disorder in a crisis.’^9^

Despite this, in routine clinical practice clearly a broad spectrum of medications is used for the symptomatic treatment of BPD, as demonstrated in Table 5, with 93% of patients prescribed at least two regular medications and more than 60% of patients on at least three regular associated medications; 87% of our BPD patients were on long-term antipsychotic medication.

Compared with the conflicting advice on medication in BPD, the international guidelines are more in agreement regarding the role of psychotherapy. APA recommends psychotherapy as the core treatment for BPD (and more than one type of psychotherapy is effective), along with adjunctive medication.^4^

There does not appear to have been a previous study of crisis or home treatment for BPD. However, a randomised trial by Bateman & Fonagy^11^ examining the effectiveness of partial hospitalisation on BPD showed that those who received individual or group psychoanalytic psychotherapy for 18 months had a significant fall in their symptoms (namely, frequency of suicide attempts, self-harm, the number and duration of hospital admissions, depressive symptoms and interpersonal functioning) by 6 months, with continued improvement until the end of treatment at 18 months. In contrast, their control group (standard psychiatric care) showed limited change or even deterioration over the same period.^11^

Our observational study showed that patients with BPD are frequent users of psychiatric services, and that some but not all respond well to home treatment as an alternative to hospitalisation. Indeed, the high re-admission rate for BPD suggests that hospital-based care is not always helpful. Despite the frequent crises experienced by this population and associated risks, there is a dearth of evidence on the best psychological and pharmacological treatments for BPD. More high-quality large studies in this area are needed.
